# Solid Pseudopapillary Tumor of the Pancreas in a Patient With Sarcoidosis: A Rare Combination

**DOI:** 10.7759/cureus.8340

**Published:** 2020-05-28

**Authors:** Ishani Shah, Harsh Mehta, Ava Anklesaria, Kambiz Kadkhodayan

**Affiliations:** 1 Internal Medicine, Creighton University School of Medicine, St. Joseph's Hospital and Medical Center, Phoenix, USA; 2 Internal Medicine, Saint Barnabas Medical Center, Livingston, USA; 3 Gastroenterology, Maimonides Medical Center, Brooklyn, USA; 4 Gastroenterology, Creighton University School of Medicine, St. Joseph's Hospital and Medical Center, Phoenix, USA

**Keywords:** pseuodpapillary tumor, pancreas, sarcoidosis, malignancy

## Abstract

Sarcoidosis is a chronic granulomatous disease that is characterized by the formation of non-caseating granulomas, predominantly involving the lung and lymph nodes. Over the years, sarcoidosis has been associated with a high risk of malignancy. Solid pseudopapillary tumor of the pancreas is an uncommon pancreatic tumor with a 15% malignant potential. Ours is an interesting case of a 34-year-old patient who was found to have a pancreatic mass and incidental mediastinal lymphadenopathy on imaging, initially raising concern for metastatic pancreatic cancer. However, she was later diagnosed to have an isolated solid pseudopapillary tumor of the pancreas in association with concurrent sarcoidosis.

## Introduction

Sarcoidosis, historically known as Besnier-Boeck-Schaumann disease, is a systemic disorder of an unclear etiology. Histologically, it is characterized by the presence of a mononuclear cellular infiltrate leading to the formation of non-caseating granulomas, mostly affecting the lungs and intra-thoracic lymph nodes in 90% to 95% of cases [1]. Almost any organ system may be involved, including the gastrointestinal tract, with the liver (5-15%) being the most commonly affected organ [1,2]. Sarcoidosis and local sarcoid-like reactions have been associated with various types of malignancies for decades. Although frequently seen in patients with various solid tumors, sarcoidosis is rarely associated with pancreatic tumors. We present a rare case of pseudopapillary tumor of the pancreas in a patient with sarcoidosis.

## Case presentation

A 34-year-old Caucasian woman with no significant medical history presented with right flank pain, low-grade fever, nausea, anorexia, and unintentional weight loss (~5 kg) for a period of two months. She denied any history of recent travel or exposure to sick contacts. On physical examination, she was afebrile with stable hemodynamics. Abdominal examination revealed tenderness to palpation in the right upper quadrant and epigastric regions. There was no costovertebral tenderness. Cardiopulmonary examination was within normal limits.

**Figure 1:**
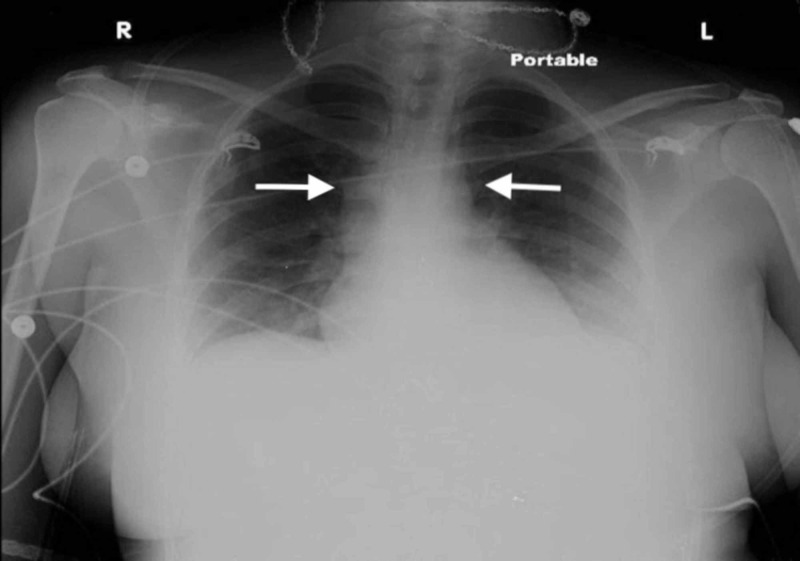
Chest X-ray showing mediastinal lymphadenopathy (white arrows) and bilateral pleural effusions.

Laboratory studies including a complete blood count, markers of liver, kidney, and thyroid function, and urinalysis were within normal limits. HIV antibody test was negative. An ultrasound of the abdomen showed a well-defined complex solid-cystic mass in the neck of the pancreas. Tumor markers (CEA [carcinoembryonic antigen], CA [cancer antigen] 19-9, and AFP [alpha fetoprotein]) were within normal limits. Additionally, a routine chest X-ray revealed suspicious mediastinal lymphadenopathy (Figure 1). CT scans of the chest and abdomen were subsequently sought that revealed mediastinal lymphadenopathy, bilateral pleural effusions, and a 4.5 x 5.5 cm mass in the neck of the pancreas with mixed solid and cystic features.

 

**Figure 2:**
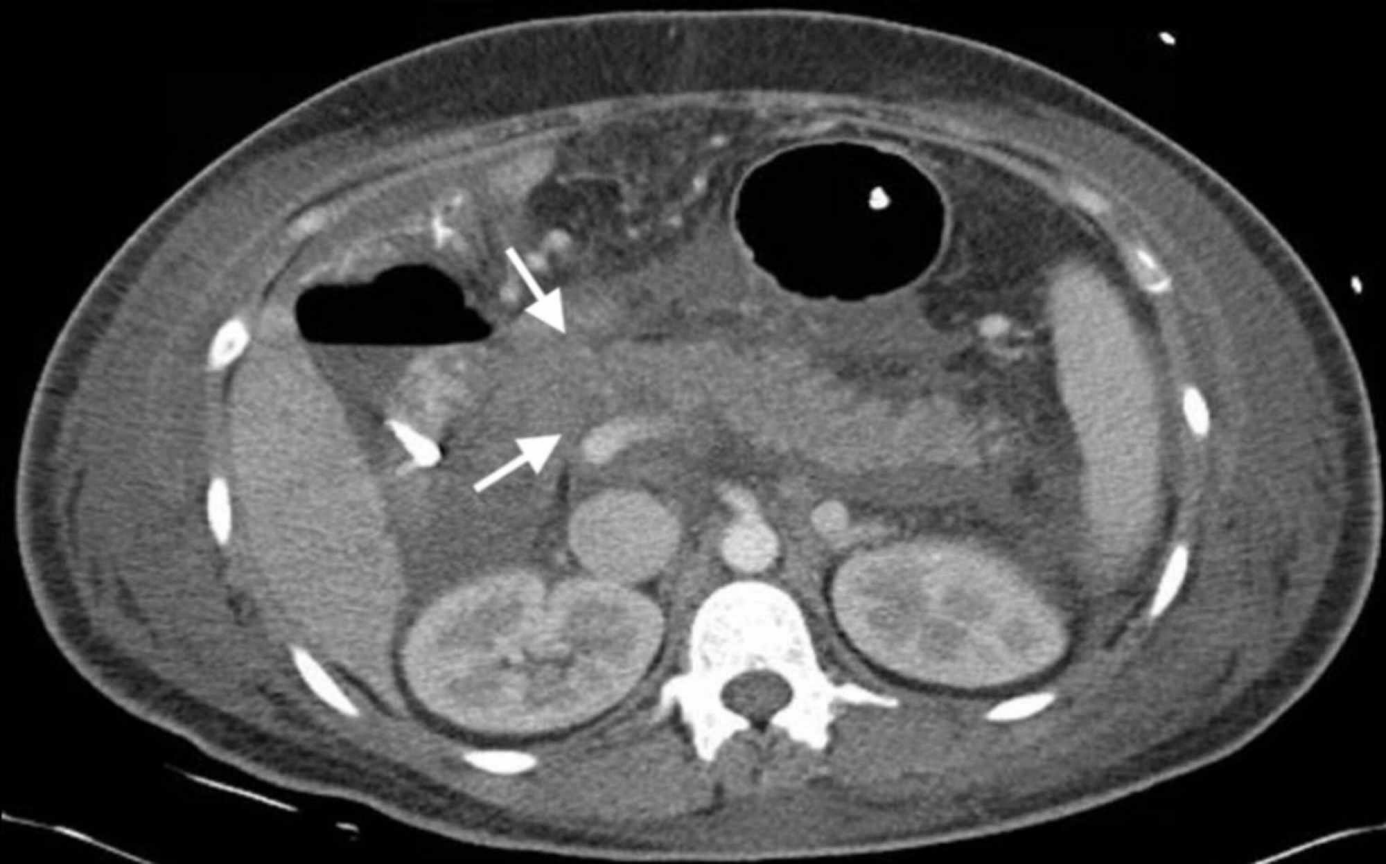
CT scan of the abdomen with post-surgical changes of pancreaticoduodenal resection (white arrows).

With the suspicion of advanced stage 4 pancreatic cancer metastatic to the lung, a left-sided mediastinoscopy and lymph node biopsy were performed to further investigate the mediastinal lymphadenopathy. Histopathology revealed multiple non-caseating granulomas. Flow cytometry and special stains (periodic acid-Schiff, Ziehl-Neelsen, Giemsa, Grocott) for microorganisms were found to be negative. On further investigation, the patient was also found to have elevated angiotensin-converting enzyme level. These findings eventually led to the diagnosis of sarcoidosis in this patient. CT-guided biopsy of the pancreatic mass revealed polygonal epithelial cells with characteristic pseudopapillary changes, leading to the diagnosis of pseudopapillary tumor (Frantz tumor) of the pancreas. Special stains including vimentin and beta-catenin were positive, thus confirming our diagnosis. Over the next few days, the patient underwent a Whipple pancreaticoduodenectomy for her pancreatic mass (Figure 2). She recovered well post-operatively and was then discharged home in a stable hemodynamic condition.

## Discussion

Sarcoidosis is a multisystem chronic granulomatous disease of unclear etiology that is prevalent in more than 0.05% of the overall population. It is almost three times more common in African Americans as compared with the White population, with a female predominance among all racial groups [2,3]. Extrapulmonary involvement is mostly observed in lymph nodes (30%) and skin (25-35%), with gastrointestinal involvement seen only in 0.1% to 0.9% of all cases [2,4]. Sarcoidosis has been associated with malignancy for several decades, with some studies reporting as high as 20% risk of developing cancer among patients with sarcoidosis [3,5,6]. However, there exist little data in the literature explaining a causal relationship between the two. Various settings have been described for this association. This includes the development of sarcoidosis-lymphoma syndrome, which manifests as lymphoma and other hematological malignancies a few years after the diagnosis of sarcoidosis. Some patients with sarcoidosis develop various solid tumors, most commonly involving the lung, liver, and skin. Similarly, many patients with preexisting cancer develop sarcoidosis as a paraneoplastic syndrome, more commonly when the diagnosis of cancer has been fairly recent. Lastly, many patients with solid tumors develop “sarcoid-like reactions”, which commonly involve regional lymph nodes and sometimes the skin [7]. To date, very few cases exist in the literature on the occurrence of pancreatic cancer in patients with sarcoidosis [8].

Solid pseudopapillary tumors of the pancreas, first described by Frantz in 1959, are rare pancreatic tumors predominantly involving the tail of the pancreas [9]. These tumors have a predilection for Asian, African American, and Indian women in the second and third decades of their lives, although they have occasionally been reported in men and children. According to a retrospective case series, pseudopapillary tumors accounted for around 3% of all resected pancreatic cystic neoplasms over a period of more than 20 years [10]. Owing to a malignant potential of around 15%, it is recommended that solid pseudopapillary pancreatic tumors should be resected in most cases. With complete resection, five-year survival rate is as high as 97% even with metastatic disease [11,12]. Interestingly, this is the second case in the literature of pseudopapillary pancreatic tumor presenting in association with sarcoidosis [13].

## Conclusions

In conclusion, sarcoidosis is associated with a high risk of malignancy and should prompt a comprehensive workup in suspicious cases. Interestingly, this is the second case in the literature of pseudopapillary pancreatic tumor presenting in association with sarcoidosis. Further studies aimed at establishing a causal relationship between sarcoidosis and malignancy are necessitated to provide more robust data.
